# Assessment of image quality on color fundus retinal images using the automatic retinal image analysis

**DOI:** 10.1038/s41598-022-13919-2

**Published:** 2022-06-21

**Authors:** Chuying Shi, Jack Lee, Gechun Wang, Xinyan Dou, Fei Yuan, Benny Zee

**Affiliations:** 1 Division of Biostatistics, Centre for Clinical Research and Biostatistics, Jockey Club School of Public Health and Primary Care, Faculty of Medicine, The Chinese University of Hong Kong, New Territories, Hong Kong, China; 2 Department of Ophthalmology, Zhongshan Hospital, Fudan University, Shanghai, China; 3 Department of Ophthalmology, Wusong Hospital, Shanghai, China

**Keywords:** Health care, Medical research, Engineering

## Abstract

Image quality assessment is essential for retinopathy detection on color fundus retinal image. However, most studies focused on the classification of good and poor quality without considering the different types of poor quality. This study developed an automatic retinal image analysis (ARIA) method, incorporating transfer net ResNet50 deep network with the automatic features generation approach to automatically assess image quality, and distinguish eye-abnormality-associated-poor-quality from artefact-associated-poor-quality on color fundus retinal images. A total of 2434 retinal images, including 1439 good quality and 995 poor quality (483 eye-abnormality-associated-poor-quality and 512 artefact-associated-poor-quality), were used for training, testing, and 10-ford cross-validation. We also analyzed the external validation with the clinical diagnosis of eye abnormality as the reference standard to evaluate the performance of the method. The sensitivity, specificity, and accuracy for testing good quality against poor quality were 98.0%, 99.1%, and 98.6%, and for differentiating between eye-abnormality-associated-poor-quality and artefact-associated-poor-quality were 92.2%, 93.8%, and 93.0%, respectively. In external validation, our method achieved an area under the ROC curve of 0.997 for the overall quality classification and 0.915 for the classification of two types of poor quality. The proposed approach, ARIA, showed good performance in testing, 10-fold cross validation and external validation. This study provides a novel angle for image quality screening based on the different poor quality types and corresponding dealing methods. It suggested that the ARIA can be used as a screening tool in the preliminary stage of retinopathy grading by telemedicine or artificial intelligence analysis.

## Introduction

Image quality, in real-world settings, is a significant aspect for diagnosis because the proportion of poor-quality images has been reported to reach up to 19.7% in non-mydriatic retinal photography^1^. Poor images are the main reason for decreasing the accuracy of retinopathy detection^2^. It has been investigated for decades to improve the automatic mage quality assessment, such as clarity assessment techniques including spatial techniques^3–11^ and wavelet transform (WT) techniques^12–16^. However, despite low computational complexity, traditional methods requiring human intervention only can identify some characteristics of image quality and have poor generalization to other datasets. Recently, some research focused on using the deep learning (DL) approach to assess image quality on color fundus retinal images. As the most popular deep learning architecture, a deep convolutional neural network (CNN) can automatically identify and extract hidden or latent features inherent in the input images with no need to define hand-crafted features^17^, and shows superior performance than traditional machine learning methods. The main studies about CNN method are summarized in the following paragraphs and Table 1.

**Table 1:** Review of studies using CNN method to assess retinal image quality.

Author	Year	Database	Method (architecture)	Category of image quality	Definition of classification	Performance
Mahapatra et al.^18^	2016	A DR screening initiative	CNN, and local saliency map	Gradable, ungradable	NA	Se: 98.2%, Sp: 97.8%, Acc: 97.9%
Yu et al.^19^	2017	Kaggle	CNN(Alexnet), and saliency map	Good, poor	NA	Se: 96.63%, Sp: 93.10%, Acc: 95.42%
Saha et al.^20^	2017	EyePACS	CNN (Alexnet)	Accept, reject, ambiguous	Yes	Acc: 100%
Zago et al.^21^	2018	DRIMDB and ELSA-Brasil	CNN (Inception-v3)	Good, poor, outlier	NA	DRIMDB: Se: 97.10%, Sp:100.0%, AUC: 99.98% ELSA-Brasil: Se: 92.00%, Sp: 96.00%,AUC: 98.56%
Chalakkala et al.^22^	2019	DRIMDB, DR1–DR2, HRF, MESSIDOR, UoA-DR, Kaggle, IDRiD,	Six pre-trained CNN (AlexNet, GoogLeNet, ResNet50, ResNet101, Inception-v3, SqueezeNet)	MSRI high quality, low quality	Some databases	Se: 98.38%; Sp: 95.19%; Acc: 97.47%
Shen et al.^23^	2020	Shanghai Diabetic Retinopathy Screening Program	Multi-task deep learning framework (VGG16)	Gradable, ungradable	Yes	Se: 83.62%, Sp: 91.72%, AUC: 94.55%
Yuen et al.^24^	2021	Primary dataset: CUHK Eye Center, National University Hospital External dataset: Hong Kong Children Eye Study, Queen’s University Belfast	Two CNN (EfficientNet-B0, MobileNetV2)	Gradable, ungradable	Yes	Se: 92.1%, Sp: 98.3%, Acc: 92.5%, AUC: 97.5%

*DL* deep learning, *CNN* convolutional neural network, *MSRI* medically suitable retinal image, *NA* not available, *Se* sensitivity, *Sp* specificity, *Acc* accuracy, *AUC* area under the curve.

In 2016, Mahapatra et al.^18^ combined unsupervised information from saliency maps and supervised information from CNN with 5 convolution layers to assess image quality. A dataset from the diabetic retinopathy (DR) screening initiative with 9653 ungradable retinal images and 11,347 gradable images was used and graded by human graders. In 2017, Yu et al.^19^ used a dataset including the training set with 3000 images and the test set with 2200 images from the Kaggle database labelled by the professionals. They extract CNN-based features as well as the unsupervised saliency map-based features and fuse them. In 2017, Saha et al.^20^ trained a deep learning framework using 3428 images (3179 accept, 249 reject, and 147 ambiguous), and the rest 3425 images (3302 accept and 123 reject) were used for evaluation. All the images were labelled by three retinal image analysis experts including one ophthalmologist from the EyePACS. They defined about 2% of the images of observation discrepancy between graders as ‘ambiguous’ category and found that they confuse the network and decrease overall performance when used for training. However, these three studies did not validate the algorithm on other datasets.

In 2018, Zago et al.^21^ used a pre-trained deep neural network (Inception-v3) and selected two public databases, namely, DRIMDB(216 retinal images divided into 125 good, 69 poor, and outlier classes) and ELSA-Brasil (842 retinal images divided into 817 good and 25 poor). The intra-database cross-validation and inter-database cross-validation (train from a database and test from another database) were used to evaluate the algorithm’s performance.

In 2019, Chalakkala et al.^22^ proposed a deep learning-based approach and tested on seven public databases. They used images from one database (DR1) as the training set and the images from all the other databases as the validation set. The training set consisted of 1500 images (800 medically suitable retinal images (MSRI) high-quality and 700 low-quality), and the validation set consisted of 7007 images (5009 MSRI high-quality and 1998 low quality). The classification of image quality was separately labelled by different graders in each database.

In 2020, Shen et al.^23^ proposed a novel multi-task deep learning framework, which can evaluate the overall image quality with quality factor analysis in terms of artefact, clarity, and field definition. It has three modules: (1) multi-task multi-factor image quality assessment; (2) landmarks (optic disc and fovea) detection; (3) semi-tied adversarial discriminative domain adaptation. Dataset in this study including 18,653 retinal images (10,000 for training and 8653 for testing) from Shanghai Diabetic Retinopathy Screening Program (SDRSP) labelled by three ophthalmologists. The training dataset and testing dataset were from different districts in Shanghai.

In 2021, Yuen et al.^24^ developed two DL-based algorithms (EfficientNet-B0 and MobileNet-V2) using 21,348 retinal photographs (14,422 in the primary dataset and 6926 in the external dataset). In addition, one ophthalmologist and two medical students labelled images targeting image quality, a field of view, and the laterality of the eye. For image quality, they achieved AUC of 0.975, 0.999, and 0.987 in the internal dataset and external 1 and 2 datasets, respectively.

In these studies, images were divided into predefined categories, usually binary classification according to whether retinal images can be used for assessment. However, among images with poor quality, except for technical failures (e.g., small pupil and wrong position of patients), some eye diseases in the late stage (e.g., mature cataract) are also noticeable causes of poor image quality^25^.

Some other studies using the automatic method in detecting retinopathy also considered the influence of image quality. Most of them manually excluded images with poor quality^2,26–30^ or created a classification model to select images with good quality^31^. However, an eye disease that can make images ungradable is usually very severe in clinic, such as central retinal vein occlusion (CRVO), or in the late stage, such as mature cataract and proliferative diabetic retinopathy (PDR). Grouping them as poor quality images will result in misdiagnosis for retinopathy and prompting treatment. Therefore, those images need to be detected in the image quality assessment stage and then suggest patients with those images be referred to ophthalmologists. Only a few studies focused on this issue. Among them, two studies put all the ungradable images into the referral category^32,33^. Another study suggested adding the visual acuity assessment into retinal analysis in DR screening, and those with reduced vision should be given a referral^34^. Therefore, if automatic image quality assessment includes differentiation between eye-abnormality-associated-poor-quality and artefact-associated-poor-quality, it would neither overlook the eye diseases with the need of a referral, nor lead to the waste of medical resources for additional examinations and invalid referral.

On the other hand, in terms of technique, images with eye-abnormality-associated-poor-quality should also be detected. There are two options to make images with poor quality gradable:

1. A recapture of images^23,35^.
2. Using artificial methods to improve image quality^36^.

However, neither of them is effective for quality improvement of eye-abnormality-associate-poor-quality. For the first option, recapturing images can only fix the technical problem of photography rather than changing the ocular structure. Thus, the repeated image acquisition for eye abnormality-associate-poor-quality leads to a waste of time, manpower, and medical resource. For the second option, even though researchers have successfully investigated and developed artificial methods to eliminate artefacts, they found that images with eye-abnormality-associated-poor-quality (e.g., asteroid hyalosis) could not be improved by those methods and remained ungradable^36^.

To the best of our knowledge, no study focuses on the automatic image analysis method in identifying eye abnormalities that contribute to the poor quality of the retinal image. In previous work, we developed an automatic retinal imaging analysis (ARIA) approach to assess the risks of stroke and cardiovascular disease (CVD) and identify lesion patterns in retinopathy^37–40^. It showed great performance in detecting patterns and extracting the data mainly through fractal analysis, high order spectra analysis and statistical texture analysis. This study aims to train and validate ARIA to automatically assess image quality and distinguish eye-abnormality-associated-poor-quality from artefact-associated-poor-quality on color fundus retinal images.

## Method

### Primary dataset

**Figure 1:**
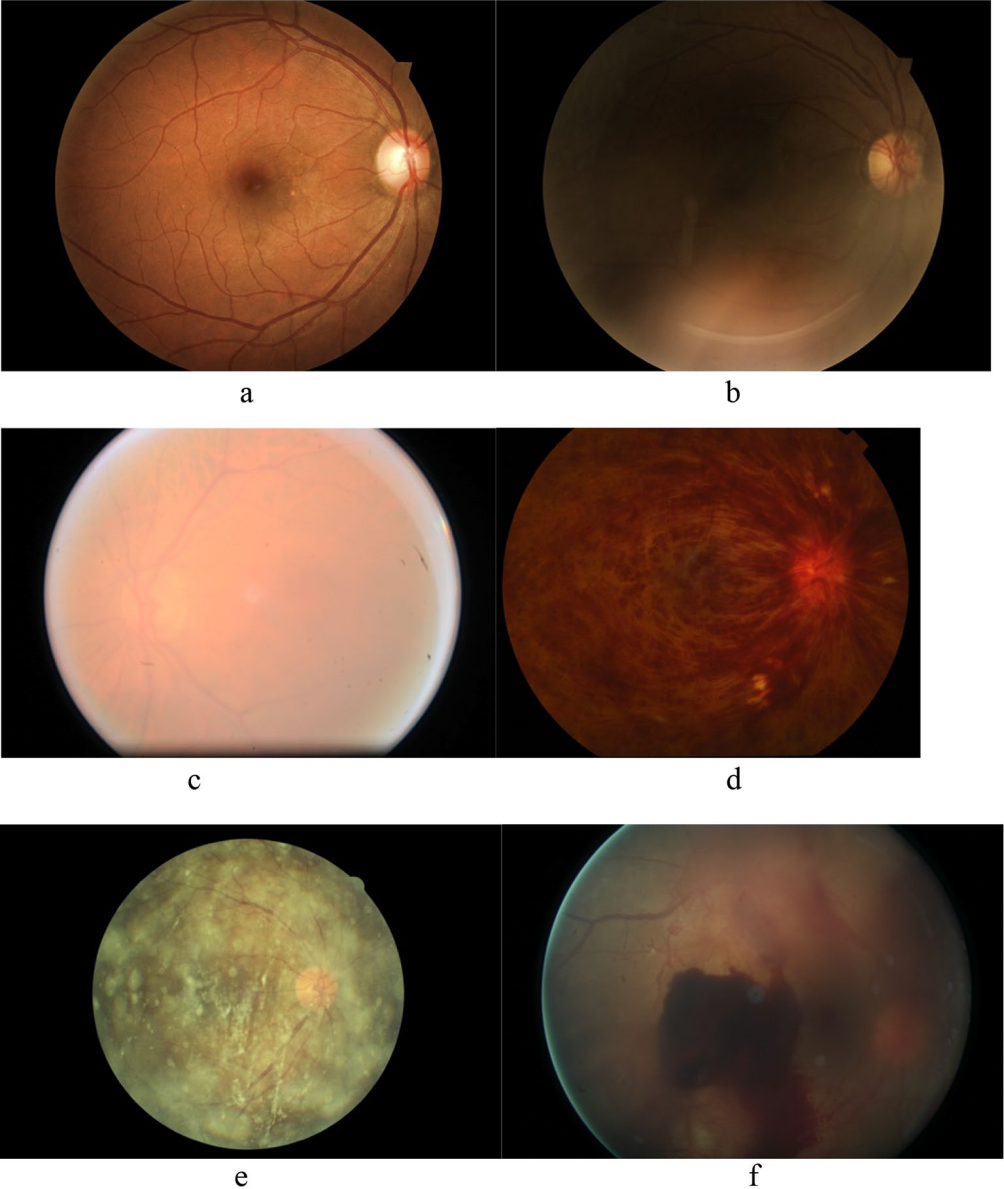
Examples of (**a**) good quality, (**b**) artefact-associated-poor-quality, and eye-abnormality-associated-poor-quality including (**c**) cataract, (**d**) central retinal vein occlusion (CRVO), (**e**) asteroid hyalosis, and (**f**) vitreous opaque.

Kaggle database, a publicly available database, was used for training and testing. The images in the dataset come from different types of cameras under various illumination. This data set contains 35,126 training images and 53,576 testing images, and many uninterpretable images with poor quality under different circumstances. Although most of the images were derived from the Kaggle database, we added a subgroup of 118 images with CRVO collected from Zhongshan hospital of Fudan University using TOPCON TRC-50DC Retinal Camera into the primary dataset because the Kaggle database lacks images with CRVO. This common retinal disease can make images ungradable. Two thousand four hundred and thirty four color fundus retinal images composed the primary dataset (Table 2). Among them, 1439 images were labelled as good quality, and 995 images were labelled as poor quality. Among images of poor quality, 483 were labelled as eye-abnormality-associated-poor-quality, and 512 as artefact-associated-poor-quality. Eye abnormalities included 220 cataracts, 118 CRVO, 83 asteroid hyalosis, and 62 vitreous opaque (Fig. 1).

**Table 2:** Summary of images in the primary dataset used for training, testing, and 10-fold cross validation.

Category	Subgroup	Number n (%)	Subgroup n (%)	Total n (%)
Good				1439 (59.12)
Poor	Eye-abnormality-associated-poor-quality		483 (19.84)	995 (40.88)
	Cataract	220 (9.04)		
	CRVO	118 (4.85)		
	AH	83 (3.41)		
	VO	62 (2.55)		
	Artefact-associated-poor-quality		512 (21.04)	
Total				2434 (100)

*CRVO* central retinal vein occlusion, *AH* Asteroid hyalosis, *VO* Vitreous opaque.

### Image quality classification and labelling

Our study's definition of image quality was evaluated according to two clinical established aspects: visibility and clarity. Images were labelled as poor quality if artefacts or eye abnormalities cover more than 1/4 of images or level III vascular arches or larger vascular arches are invisible (Table 3) based on the recommendation of the threshold of image quality^23^. Among images with poor quality, they were categorized into eye-abnormality-associated-poor-quality and artefact-associated-poor-quality. Three ophthalmologists rated all the images in the training and external validation datasets; all of them have more than 5 years of experience. If there was a discordance result between ophthalmologists, another ophthalmologist with experience over ten years would make a final decision.

**Table 3:** Definition of image quality classification.

Image quality	Visibility	Clarity
Good	Artefacts or eye abnormalities cover less than 1/4 of image	Level III vascular arches are visible
**Poor**		
Eye abnormality associated	Eye abnormalities cover more than 1/4 of image	Level III vascular arches or larger vascular arches are invisible
Artefact associated	Artefacts cover more than 1/4 of image	Level III vascular arches or larger vascular arches are invisible

### External validation dataset

We used images from the Zhongshan Hospital of Fudan University for ARIA external validation. This study and data collection were approved by the Research Ethics Committee of Zhongshan Hospital and were performed according to the tenets of the Declaration of Helsinki. Images were captured by TOPCON TRC-NW100 Non-mydriatic Retinal Camera and TOPCON TRC-50DC Retinal Camera. It is worth mentioning that there was no overlap of selected images from the same eyes between the two cameras. Three hundred and fifty six color fundus retinal images composed the validation dataset (198 good quality, and 158 poor quality), and show on Table 4. Among images with poor quality, 104 were labelled as eye-abnormality-associated-poor-quality, and 54 as artefact-associated-poor-quality. Eye abnormality included 56 cataracts, 32 CRVO, and 16 vitreous opaque. The definition of image quality classification was the same as the training and testing dataset. The labels of specific eye abnormalities were the clinical diagnosis confirmed by ophthalmologists.

**Table 4:** Summary of images in the external validation dataset taken by TOPCON TRC-NW100 non-mydriatic retinal camera and TOPCON TRC-50DC retinal camera from the hospital.

Category	Subgroup	Number n (%)	Subgroup total n (%)	Total n (%)
Good				198 (55.62)
Poor	Eye-abnormality-associated-poor-quality		104 (29.21)	158 (44.38)
	Cataract	56 (15.73)		
	CRVO	32 (8.99)		
	VO	16 (4.49)		
	Artefact-associated-poor-quality		54 (15.17)	
Total				356 (100)

*CRVO* central retinal vein occlusion, *VO* Vitreous opaque.

### Training, testing, and external validation

In the primary dataset, the model(s) building with 70% of images was for training and 30% for testing. The study included deep learning models for (1) classification of all good quality and all poor quality images, (2) classification of all good quality and eye-abnormality-associated-poor-quality, (3) classification of all good quality and artefact-associated-poor-quality and (4) classification of eye abnormality-associated poor quality and artefact-associated-poor-quality.

**Figure 2:**
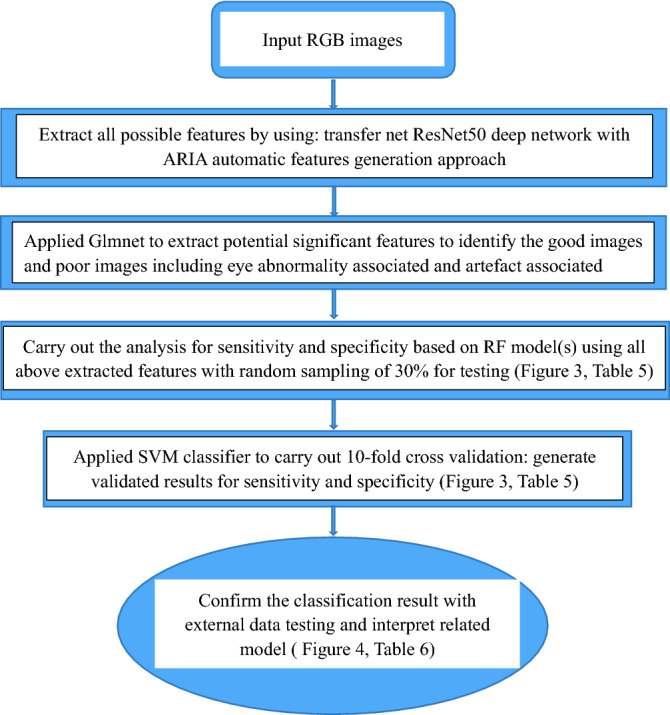
Flowchart of the presented method in image quality classification. *ResNet50* Residual network that is 50 layers deep, *Glmnet* Generalized linear model via penalized maximum likelihood, *RF* Random forest, *SVM* Support vector machine.

We used the automatic retinal image analysis(ARIA) method developed using Matlab, which has been reported (US Patent 8787638 B2)^41^. It incorporates deep learning techniques to classify image quality and shows on Fig. 2. Firstly, we applied transfer net ResNet-50 deep network with retinal images (RGB) as input, and features generated at the layer of ''fc1000_softmax'' as output based on pixels associated with image quality^42^. We also used the ARIA automatic features generation approach to extract the texture/fractal/spectrum-related features written in Matlab^41^. Then we applied the Glmnet approach to select the most important subset of features based on the penalized maximum likelihood^43^. These refined features are highly associated with image quality and were used to generate models by random forest (RF) in Matlab. The rest, 30% of the primary dataset, was used to test the performance of models. After internal portion testing and the model built, we also applied a tenfold cross-validation with support vector machine (SVM) approach for the model assessment in order to avoid over-classification. Finally, we confirmed the ability of models to classify image quality in the external validation dataset and analyzed the false positive cases and false-negative cases.

### Statistical analysis

The performance of prediction models in training and testing dataset by calculating sensitivity, specificity, and accuracy from the testing dataset and 10-fold crossing validation. In external validation, the area under the curve (AUC) of receiver operator characteristic (ROC) curve of analysis, sensitivity, specificity, accuracy, and the proportion of the false-positive cases and false-negative cases of external validation were calculated. All analyses were performed using the software of SPSS 20 and Matlab 2020a.

## Result

**Figure 3:**
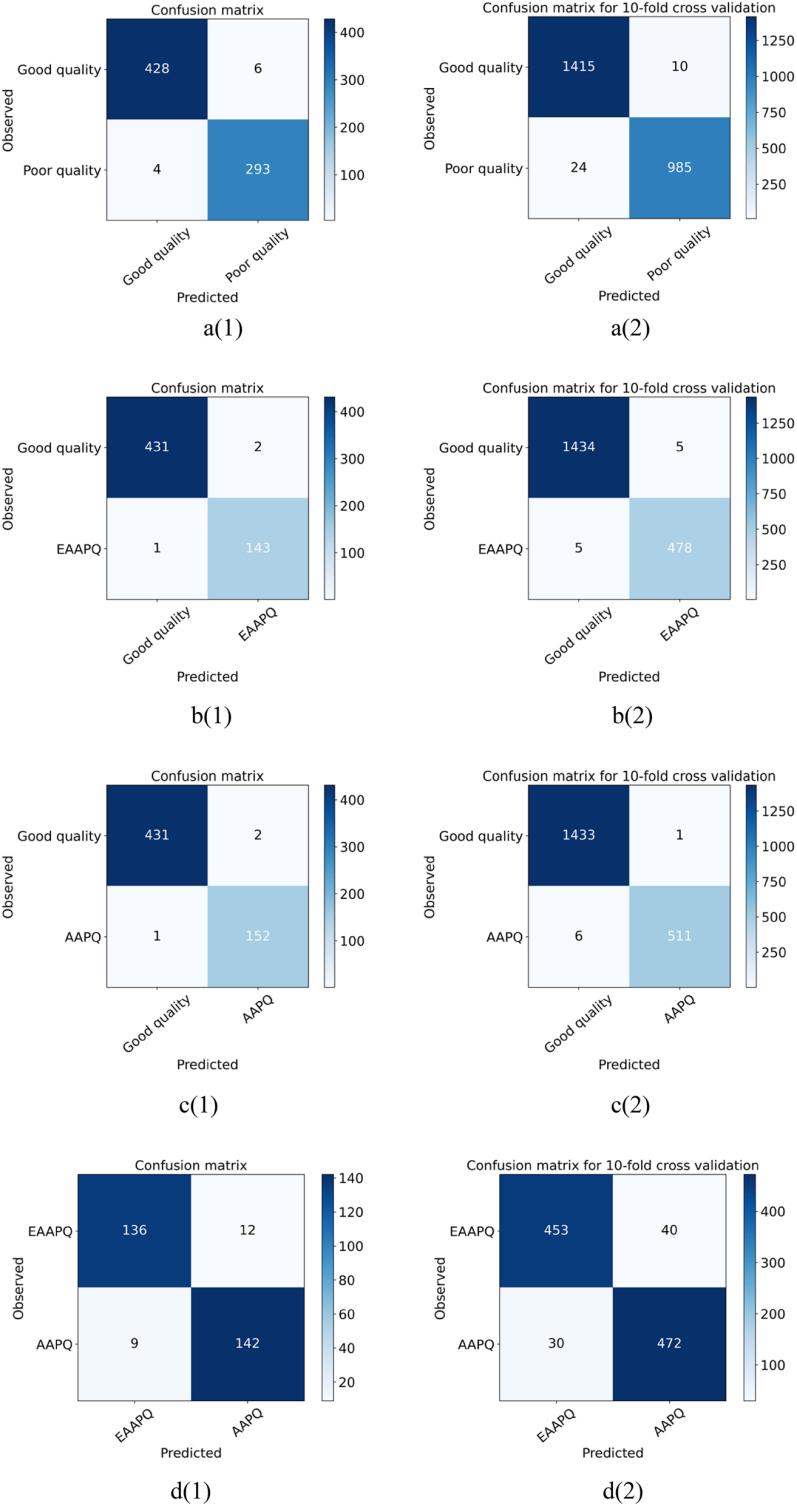
Confusion matrix of testing (1) and 10-fold cross validation (2). (**a(1)**, **a(2)**) Good quality VS Poor quality; (**b(1)**, **b(2)**) Good quality VS Eye-abnormality-associated-poor-quality; (**c(1)**, **c(2)**) Good quality VS Artefact-associated-poor-quality; (**d(1)**, **d(2)**) Eye-abnormality-associated-poor-quality VS Artefact-associated-poor-quality.

The performance of ARIA in differentiating different categories of image quality was summarized on Table 5 and Fig. 3. Using a simple random sampling method, 1007 images with good quality and 696 images with poor quality (including 338 images with eye-abnormality-associated-poor-quality and 358 images with artefact-associated-poor-quality) were assigned to the training dataset. The remaining 432 images with good quality and 299 images with poor quality (including 145 images with eye-abnormality-associated-poor-quality and 154 images with artefact-associated-poor-quality) were held out for testing. The sensitivity, specificity and accuracy of the ARIA for testing good quality against poor quality were 98.0%, 99.1%, and 98.6%, and ones in tenfold cross-validation were 99.0%, 98.3%, and 98.6%, respectively. The sensitivity, specificity and accuracy of the ARIA for testing good quality VS eye-abnormality-associated-poor-quality were 98.6%, 99.8%, and 99.5%, and ones in tenfold cross-validation were 99.0%, 99.7%, and 99.5%, respectively. The sensitivity, specificity and accuracy of the ARIA for testing good quality against artefact-associated-poor-quality were 98.7%, 99.8%, and 99.5%, and ones in tenfold cross-validation were 99.8%, 99.6%, and 99.6%, respectively. Last but not least, the sensitivity, specificity and accuracy of the ARIA for testing eye-abnormality-associated-poor-quality against artefact-associated-poor-quality were 92.2%, 93.8%, and 93.0%, and ones in tenfold cross-validation were 92.2%, 93.8%, and 93.0%, respectively.

**Table 5:** The performance of ARIA in testing dataset.

	Testing (using RF)			Ten-fold cross validation (using SVM)		
	Se (%)	Sp (%)	Acc (%)	Se (%)	Sp (%)	Acc (%)
Good quality VS poor quality	98.0	99.1	98.6	99.0	98.3	98.6
Good quality VS EAAPQ	98.6	99.8	99.5	99.0	99.7	99.5
Good quality VS AAPQ	98.7	99.8	99.5	99.8	99.6	99.6
EEAPQ VS AAPO	93.8	92.2	93.0	93.8	92.2	93.0

*RF* Random forest, *SVM* Support vector machine, *EAAPQ* Eye-abnormality-associated-poor-quality, *AAPQ* Artefact-associated-poor-quality, *Se* Sensitivity, *Sp* Specificity, *Acc* Accuracy.

**Figure 4:**
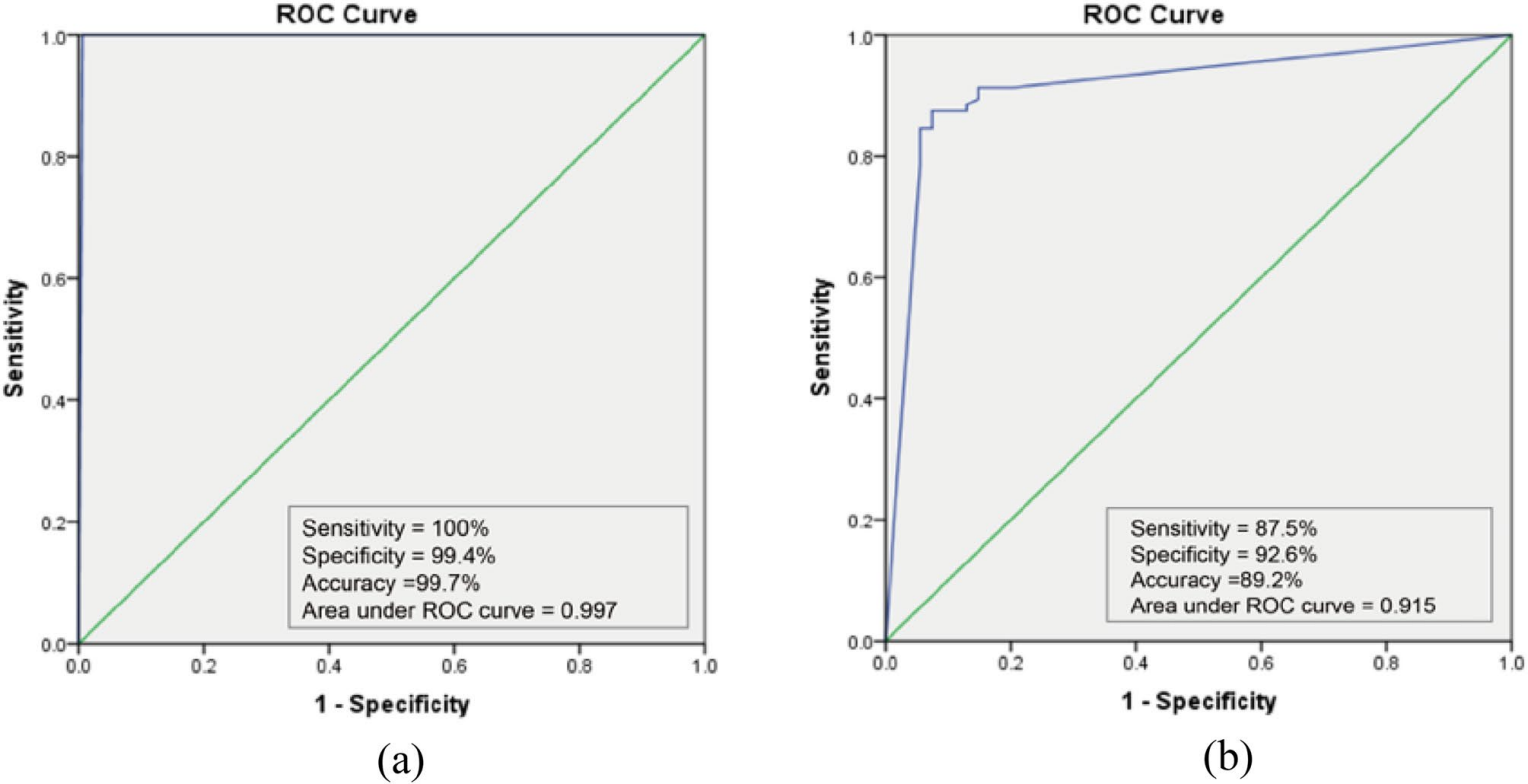
(**a**) The performance of ARIA to differentiate good quality from poor quality in the external validation dataset; (**b**) The performance of ARIA to differentiate artefact-associated-poor-quality from eye-abnormality-associated-poor-quality in the external validation dataset.

**Figure 5:**
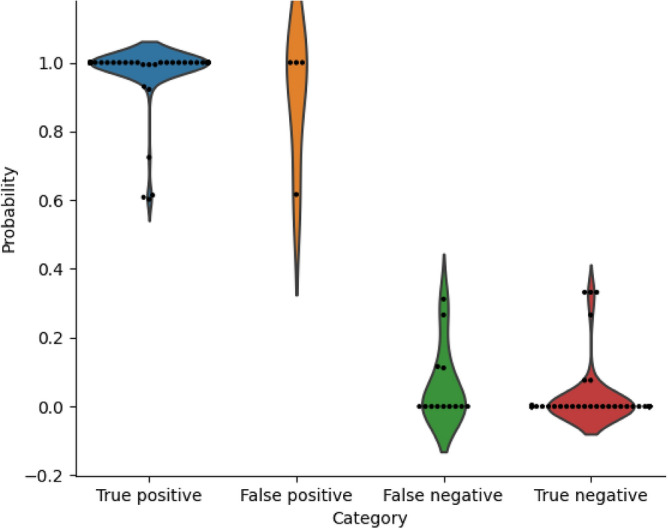
Violin plot for the probability of ARIA output in differentiating artefact-associated-poor-quality from eye-abnormality-associated-poor-quality in the external validation dataset (predicted value of probability: 0: Artefact-associated-poor-quality; 1: Eye-abnormality-associated-poor-quality).

In the external validation, ARIA also showed comparable and robust results in distinguishing between good quality and poor quality (Fig. 4a), achieving a sensitivity of 100%, the specificity of 99.4%, the accuracy of 99.7%, and the area under the ROC curve of 0.997. The performance of ARIA was also evaluated in distinguishing between artefact-associated-poor-quality and eye-abnormality-associated-poor-quality (Fig. 4b). The sensitivity, specificity, accuracy, and area under the ROC curve were 87.5%, 92.6%, 98.2%, and 0.915, respectively. Furthermore, details of misclassification of artefact-associated-poor-quality and eye-abnormality-associated-poor-quality were shown on Table 6 and Fig. 5. Eye abnormalities of false-negative cases included CRVO (n = 7) and vitreous opaque (n = 6), which showed that these images with these eye abnormalities were misclassified as images with artefacts.

**Table 6:** The misclassification of ARIA in differentiating artefact-associated-poor-quality from eye-abnormality-associated-poor-quality.

	No	Proportion (%)
**False-positive images**		
Blur	3	75.0
Underexposure	1	25.0
Total	4	100.0
**False-negative images**		
CRVO	7	53.8
Vitreous opaque	6	46.2
Total	13	100.0

*CRVO* central retinal vein occlusion.

## Discussion

In this study, we trained and validated an automatic method, ARIA, to automatically assess image quality. In evaluating color retinal images from a multiethnic public dataset and an external dataset from the hospital, the ARIA had excellent performance in classifying good quality and poor quality compared to the performance of other automatic methods (Table 1). Additionally, the differentiation of two types of poor quality is more challenging than the classification of good and poor quality and did not investigate before. Our results also show that the ARIA can differentiate eye-abnormality-associated-poor-quality from artefact-associated-poor-quality. Therefore, this approach has the potential to be used as a screening tool for automatic retinal image quality assessment in the preliminary stage of retinopathy detection. Furthermore, poor quality images with eye abnormality can be filtered out and referred to an ophthalmologist. In contrast, images with artefact-associated-poor-quality require image improvement by the second photography or image processing.

Our study included some of the most common eye abnormalities that may cause poor image quality. Media opacity has been reported to play a critical role in causing poor image quality^44–48^, and some studies focusing on automatic retinopathy grading excluded them before the training procedure^30,49^. A cataract is lens opacity, a type of the most common opacity. According to the definition of four grades of cataract^50^, cataract in the advanced stage was labelled as eye-abnormality-associated-poor-quality and successfully detected in our study. Suppose we screened out images as poor quality during the image assessment procedure. In that case, patients will lose the opportunity for prompt cataract surgery. They may lead to hyper mature senile cataract (HMSC) or even adverse complications such as lens-induced uveitis phagocytic glaucoma and rarely spontaneous dislocation of the nucleus^51–53^.

Besides cataracts, vitreous opaque, such as haemorrhage and proliferative membrane, and retinal vein occlusion (RVO) are also the common eye abnormalities that can make images ungradable and should be promptly referred to doctors in case of the complication of retinal detachment, retinal tears, neovascular event, and retinal capillary nonperfusion^54,55^, which may lead to permanent vision loss or blindness. In addition, in external validation, we found that CRVO and opaque vitreous consist of the entire false-positive cases. It indicates that they are more likely to be misclassified as artefact-associated-poor-quality compared to cataracts, probably due to the relatively small amount of training images and various patterns of lesions on images. This investigation will be helpful for a better understanding of ARIA grading. In the future, to optimize the model, more images with CRVO and vitreous opaque can be included in the training dataset.

Asteroid hyalosis is an eye abnormality; that vitreous body contains small yellow-white, spherical particles known as asteroid bodies (ABs)^56^. Although it barely impacts on the patient’s vision, it can significantly influence the clinical examination of ophthalmologists on the fundus. Severe asteroid hyalinosis can even render fundus examination impossible^56,57^. Moreover, the estimated global prevalence of asteroid hyalinosis continues increasing, and old people make up a substantial and increasing fraction^57^. Hence, although most of those patients do not need a referral, the images should also be screened out in the quality screening stage due to the increasing prevalence and impairment of fundus assessment. It is worth mentioning that cataract, asteroid hyalinosis, and RVO are the conditions becoming increasingly prevalent with age^58,59^. Therefore, in age-related retinopathy screening programs, such as age-related macula disease (AMD), a larger proportion of eye disease is associated with poor quality, which influences the retinopathy assessment. In the above, we should pay more attention to the eye abnormalities associated with poor image quality in those programs.

## Conclusion

Our ARIA approach showed good performance in identifying eye-abnormality-associated-poor-quality and artefact-associated-poor-quality and distinguishing between good and poor quality. Thus, images with eye abnormality-associated-poor-quality can be filtered out and be referred to an ophthalmologist. On the other hand, images with artefact-associated-poor-quality denote a requirement for quality improvement by the second photography or image processing. Further research can enlarge the variety of eye diseases that can lead to poor image quality in the ARIA method and evaluate the applicability and utility of the real-world clinical practice.
